# Adhesion of the Placenta

**Published:** 1858-03

**Authors:** John W. Jones

**Affiliations:** Prof. of Principles and Practice of Medicine in the Atlanta Medical College, Atlanta, Ga.


ATLANTA

Medical and Surgical Journal.

VOL. III.] MARCH, 1858. [No. VII.

### ORIGINAL COMMUNICATIONS.

### ARTICLE 1.

Note from an old Case Book.

*Adhesion of the Placenta.* BY JOHN W. JONES, M. D., Prof. of Principles and Practice of Medicine in the Atlanta Medical College, Atlanta, Ga.

Finding in my old case book, the following note of the management of an obstetrical case, some of the particulars of which are of rare occurrence and may not prove uninteresting to the juvenile practitioner; I have thought fit to present it for your pages.

I am, by no means, unapprised of the fact that such or similar cases have occurred under the observation of others, nor do I assert that it is unique or "sui generiis." Nevertheless, I cannot but believe that a summary account of the one which it was my lot to attend, if communicated, through the medi_ um of your very interesting periodical, will not prove altogether uninteresting to the philanthropic accoucheur.

At 6 o'clock, P. M., of the 19th of May, 1846, I was summoned to attend Mrs. R. in her first accouchment; aged 30 years of middle stature, constitution ordinary—previous health moderately good. Nothing special transpired during the night, except the usual developments of incipient parturition. Early on the morning of the 20th, during each contractile effort of the uterus, (the os tincæ being considerably dilated and moderately dilatable,) I perceived a gradual advancement of the fœtal head, it being the first vertex presentation of Dewees. The bladder and intestines having been previously well evacuated, I left her to the Vis conservatrix naturæ, until noon of the same day, when, upon examination, I found the vertex at the inferior strait, and rather stationary, even under the influence of strong uterine contractions, and the parts but sparingly lubricated. I now endeavored to ascertain the state of the circulation, and found it too languid to justify venesection. The pains having continued forcible and frequent, for some two hours longer, the head was slowly protruded through the labiæ and in a few minutes thereafter, my patient was delivered of a common sized male infant, not, however, until I had removed from around its neck three coils of the umbilical cord.

The last mentioned circumstance I very naturally anticipated prior to its actual discovery, in consequence of the head having receded partially, immediately subsequent to several preceding pains. After having waited in vain for the space of twenty or thirty minutes for the expulsive efforts of the uterus to deliver the placenta, I directed frictions with the hand, over the abdomen, and simultaneously used gentle traction, by means of the cord, all of which seemed to accomplish nothing salutary.

My patient was now growing restless and rapidly becoming pale, and, also, complained of an entire loss of vision; and in a short time vomited freely and then was calm, her extremities cool and fast growing cold, pulse feeble and the entire body covered with profuse perspiration. I immediately commenced the liberal use of a decoction of capsicum, and wrapped the extremities well with sanapisms. No pain, and but little hemorrhage; alternated Cognac Brandy with the Capsicum; gave 20 grains of the Secale Cornutum, in four portions, every 15 minutes, in the form of decoction.

Sickness and emesis recurred; arterial action imperceptible at the wrist; extremities cold and clammy—face blanched, no pain; continued the stimulants, and added ten drops of the tinct Opii every 15 minutes, until 100 drops had been administered, by which time the radial artery began feebly to pulsate. She soon fell into a profound sleep and in a few hours the pulse seemed permanently strong and full.

I now had her aroused, and proceeded to manipulate so as to command the entire placenta, and endeavored, by every gentle means in my power, to extract it, which I soon found would be difficult, if not impossible, in consequence of its firm adherence to the anterior wall of the uterus. I continued my efforts faithfully for twenty or thirty minutes, and only succeeded in detaching, probably one-third of the placental mass; the remaining portion I could not separate by any effort which I conceived justifiable. I now discontinued all further interference, as my patient was again pale, pulseless and vomiting and appeared to be rapidly approximating dissolution. Nor did I do anything in the way of treatment for sometime thereafter, except endeavoruing to support the almost extinguished flame of life, which but feebly flickered in some of the more vital organs. At the same time resolving to leave the placenta to be expelled by uterine contractions or decomposition, &c., recollecting that "Ut quimus quando ut volumus non licet," and believing the "Vis medicatrix naturæ" to be "Ultimum Unicum Remedium." I therefore determined to rely upon the "Vis Vitæ," notwithstanding "Via trita, Via tuta," I resolved to depart from a practice too much followed, viz.: to remove it by manipulation.

My friend, Dr. Clark, in obedience to my request, had been sent for, and arrived about 9 o'clock, P. M., and after possessing himself of all the facts of the case, without hesitation confirmed my decision and determination, though, not until he had fully satisfied himself that the remaining portion of the placenta could not, with impunity, be detached. Dr. Clark advised the employment of stimulating enemas, within the rectum, which was accordingly attended to.

On the morning of the 21st I saw my patient and found her doing well; on the 22d all the symptoms were favorable; at 1 o'clock, P. M., of the 23d, the remainder of the placenta was discharged by the moderate and spontaneous contractions of the uterus. The nurse was directed to use injections, per vaginum, three times daily, composed of diluted Pyroligneous acid. At noon of the 28th my patient was laboring under severe headache and high arterial action, hot skin, &c. Ordered 20 grains Sub. Mur. Hyd. to be followed in 6 hours by Oleum Ricini. On the morning of the 29th I called, and learned that she had had several copious bilious evacuations, and expressed herself much relieved, continued convalescing up to this time, July 6th, and is now in the enjoyment of good health.

*Remarks.*—My object in thus submitting the outlines of this case to the medical public has been already announced. Past, and very painful experience has long since taught me the deleterious consequences of rashly separating the placenta under similar circumstances; and I am much gratified in the recollection that such a measure has never been proposed or sanctioned by me. Often, very often, have I been compelled to use manual assistance in affecting the delivery of the placenta. But sixteen years obstetrical experience has not furnished me a single example in which the placenta was so firmly adherent, or one which did not yield to persevering manipulations, except the one which constitutes the basis of this brief and very incomplete report.

When it is recollected that two-thirds of the placenta remained undetached for three days after delivery, and that there must have been great inertia of the uterus, evidenced by the absence of uterine contractions, (with the exception of tonic contraction) and pain, is it not remarkable, that the hemorrhage was so very limited !

There is another important feature of the case which merits profound attention, viz. : No perceptible excitement was produced by the use of the stimulants, (and they were active and powerful excitants,) although liberally administered for several hours, until their action was accompanied by the anodyne qualities of the opium. So soon, however, as the patient slept, the invigorating and life-sustaining qualities of the pepper and brandy, were most happily manifested, from all of which, what do we reasonably infer ?

1st. That a protracted and painful labor having terminated, all the excitement that arose from the presence of the fœtus, and the pains it excited, also, having ceased, two reasons may be assigned for the diminution of excitement: the abstraction of stimuli, and exhaustion of excitability from over action, &c. Hence, the depression, exhaustion, and collapse.

2d. That, as sleep or rest is an indispensable prerequisite to restore excitability—and as the administration of opium (as a matter of course,) was followed by sleep; and, as the excitants did not produce the desired effect, until the patient slept, we infer that Anodyne and Sedatives favor the accumulation of excitability, and that a certain degree of excitability is, also, indispensable to the action of excitants.

Hence, we discover, in certain cases, the vast importance of combining sedatives and anodynes with stimulants or excitants. With this very succinct detail of a case, (to me exceedingly interesting,) I will conclude lest I be suspected of laboring under a malady too prevalent in this, our day, viz.: *Cacœthes Scribendi.*

